# Glaucarubinone sensitizes KB cells to paclitaxel by inhibiting ABC transporters via ROS-dependent and p53-mediated activation of apoptotic signaling pathways

**DOI:** 10.18632/oncotarget.9865

**Published:** 2016-06-07

**Authors:** Subburayan Karthikeyan, Sugeerappa Laxmanappa Hoti, Yasin Nazeer, Harsha Vasudev Hegde

**Affiliations:** ^1^ Regional Medical Research Centre, Indian Council of Medical Research (ICMR), Karnataka, India

**Keywords:** multidrug resistance, glaucarubinone, paclitaxel, molecular docking, apoptosis

## Abstract

Multidrug resistance (MDR) is considered to be the major contributor to failure of chemotherapy in oral squamous cell carcinoma (SCC). This study was aimed to explore the effects and mechanisms of glaucarubinone (GLU), one of the major quassinoids from *Simarouba glauca* DC, in potentiating cytotoxicity of paclitaxel (PTX), an anticancer drug in KB cells. Our data showed that the administration of GLU pre-treatment significantly enhanced PTX anti-proliferative effect in ABCB1 over-expressing KB cells. The Rh 123 drug efflux studies revealed that there was a significant transport function inhibition by GLU-PTX treatment. Interestingly, it was also found that this enhanced anticancer efficacy of GLU was associated with PTX-induced cell arrest in the G2/M phase of cell cycle. Further, the combined treatment of GLU-PTX had significant decrease in the expression levels of P-gp, MRPs, and BCRP in resistant KB cells at both mRNA and protein levels. Furthermore, the combination treatments showed significant reactive oxygen species (ROS) production, chromatin condensation and reduced mitochondrial membrane potential in resistant KB cells. The results from DNA fragmentation analysis also demonstrated the GLU induced apoptosis in KB cells and its synergy with PTX. Importantly, GLU and/or PTX triggered apoptosis through the activation of pro-apoptotic proteins such as p53, Bax, and caspase-9. Our findings demonstrated for the first time that GLU causes cell death in human oral cancer cells via the ROS-dependent suppression of MDR transporters and p53-mediated activation of the intrinsic mitochondrial pathway of apoptosis. Additionally, the present study also focussed on investigation of the protective effect of GLU and combination drugs in human normal blood lymphocytes. Normal blood lymphocytes assay indicated that GLU is able to induce selective toxicity in cancer cells and *in silico* molecular docking studies support the choice of GLU as ABC inhibitor to enhance PTX efficacy. Thus, GLU has the potential to enhance the activity of PTX and hence can be a good alternate treatment strategy for the reversal of PTX resistance.

## INTRODUCTION

World Health Organisation (WHO) has estimated that 91% of oral cancers in South-East Asia are directly attributable to the use of tobacco and this is often the leading explanation of oral cavity and lung cancer in Asian country as well as globally [1]. It is well known that multidrug resistance (MDR) is a major obstacle in the chemotherapy of oral cancer. Clinically, several resistance proteins, including phosphoglycoprotein (P-gp), multidrug resistance associated proteins (MRPs), and breast cancer resistance protein (BCRP) are proved to be simultaneously involved in MDR of oral cancer [2]. Conventional single-agent chemotherapy treatment has potential risks of high toxicity for the reason that the high dosage of individual anticancer drugs. Adding a second agent to the standard chemotherapy treatment would be favourable because this approach might reduce the dose, toxicity and improve the anticancer efficacy of original antineoplastic agents [3]. As a result of unfavourable clinical outcome to previous MDR inhibitors, research has focused on searching for naturally occurring MDR inhibitor candidates. We recently reported that natural compound including flavonoids, such as resveratrol inhibits MDR transport function directly in ABCB1 overexpressing NCI-H460 lung cancer cells [4]. Further, we have challenged to provide a recent update of the published work on the natural products which have shown promising chemosensitizing effects on ABCs drug transporters [2]. In our continuous search of naturally occurring cytotoxic compounds, we designed this study to assess the cytotoxicity of glaucarubinone (GLU), a quassinoid natural product first isolated from the seeds of *Simarouba glauca*, and later from numerous other species in the family Simaroubaceae [5]. It was originally developed as an antimalarial drug [6]. Although GLU has been shown to possess anti-cancer activity [7, 8], the mechanisms involved are not fully understood. The leaves of this tree are being used for treatment of cancers. A recent report indicated that quassinoids, including GLU inhibited the transcription factor activator protein-1 (AP-1), independently of their cytotoxicity [9]. Several independent lines of evidence suggest that GLU may possibly act, at least in part, through inhibition of pathways involving P-21-activated kinases1 (PAK1). A very recent report shows that GLU inactivates NF-kB, whose activation requires PAK1. Furthermore, GLU is also reported to extend the lifespan of *Caenorhabditis elegans*, which is also increased by down-regulation of PAK1 [10].

Human oral squamous cell carcinoma in KB cell line highly express classical membrane transporter, P-gp in the nuclear envelope and cytoplasm in KB cells [11]. It is reported that P-gp is involved in resistance to doxorubicin, paclitaxel, vincristine and etoposide. Additionally, this cell line has been widely used to study a number of substrates and inhibitors such as P-gp, MRP and BCRP [12]. Paclitaxel (PTX) that works by intervening with normal microtubule breakdown during cell division has been one of the important successful antineoplastic drugs and has shown its potency against a wide spectrum of cancers, especially against oral, non-small cell lung cancer, metastatic breast cancer and refractory ovarian cancer [4]. The frequent development of MDR hampers the efficacy and hence use of PTX for cancer treatment. The over-expression of P-gp and MDR linked proteins such as MRP1 and MRP2, which actively efflux anticancer drugs, is one of the most important mechanisms concerned in PTX-mediated MDR [12]. This kind of typical MDR mechanism is called as “pump resistance” [13]. PTX is a substrate of P-gp, MRP1, and MRP2 [12, 4]. In addition, the capability of PTX to kill cancer cells decreases when the event of MDR. This phenomenon happens as a result of cell death is avoided via the activation of a survival factor, such as Bcl-2 protein, that is markedly over expressed in many cancer cells [14, 13]. Thus, the defect of apoptotic machinery in cancer cells leads to “non-pump resistance” [14]. The reversal action of MDR using multifunctional strategies that inhibit pump and non-pump resistance is more suitable because of the difficulty of PTX-resistance mechanisms.

Hence, there is need for modulators to increase PTX accumulation in resistant KB cells. To our knowledge yet no studies are available in literature evaluating the chemosensitizing effect of GLU and examined their ability to overcome P-gp mediated MDR in PTX resistant ABCB1 overexpressing oral carcinoma KB cells *in vitro* and *in vivo*. Hence, the scope of the present study is limited to uncover the molecular basis and a novel combination of GLU and PTX to simultaneously modulate pump and non-pump resistance for the treatment of ABCB1 over expressing in oral carcinoma KB cells *in vitro*.

## RESULTS

### Dose fixation study

**Figure 1:**
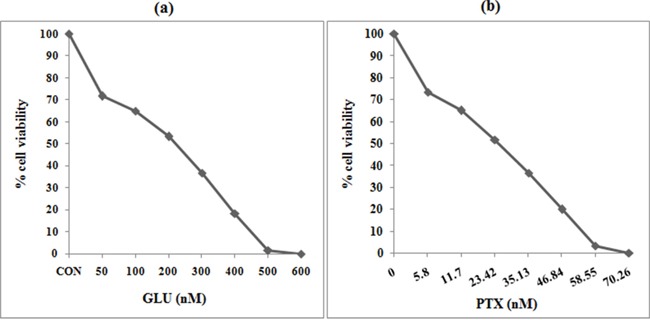
Cell viability was measured by MTT assay KB cells were plated in 96-well plates at a density of 5000 cells/well. After overnight culture, cells were treated with different concentrations of GLU or PTX or their combination for 24. All stock compounds were dissolved in DMSO which was maintained in 0.01% for drug treatments. After treatment with the compounds, 10 μL of 5 mg/mL MTT solution was added to each well and incubated for 4 h. Plates were placed on a plate shaker for 30 min and read immediately at 570 nm using an enzyme-linked immunosorbent assay reader. Experiments were performed in triplicate. **a.** Cytotoxicity of GLU on resistant KB cells (Dose fixation study). Cell death was observed in a concentration (0–600 nM) dependent manner in KB cells. 200 nM GLU showed 50% cell death (IC_50_) in KB cells. **b.** Cytotoxicity of PTX on sensitive KB cells. Cell death was observed in a concentration (0–70 nM) dependent manner in KB cells. 23.42 nM PTX showed 50% cell death (IC_50_) in KB cells.

The anti-proliferative effect of GLU was found to be concentration dependent and 100% death of resistant KB cells was observed at 600 nM of GLU concentration, while 100% death was observed at 70.26 nM of PTX concentration in sensitive KB cells. Based on these findings 200 nM of GLU (IC 50) and 23.42 nM of PTX (IC 50) were chosen for chemosensitizing experiments (Figure 1a & 1b).

### GLU and PTX combined treatment significantly intensified PTX cell viability

**Figure 2a & b:**
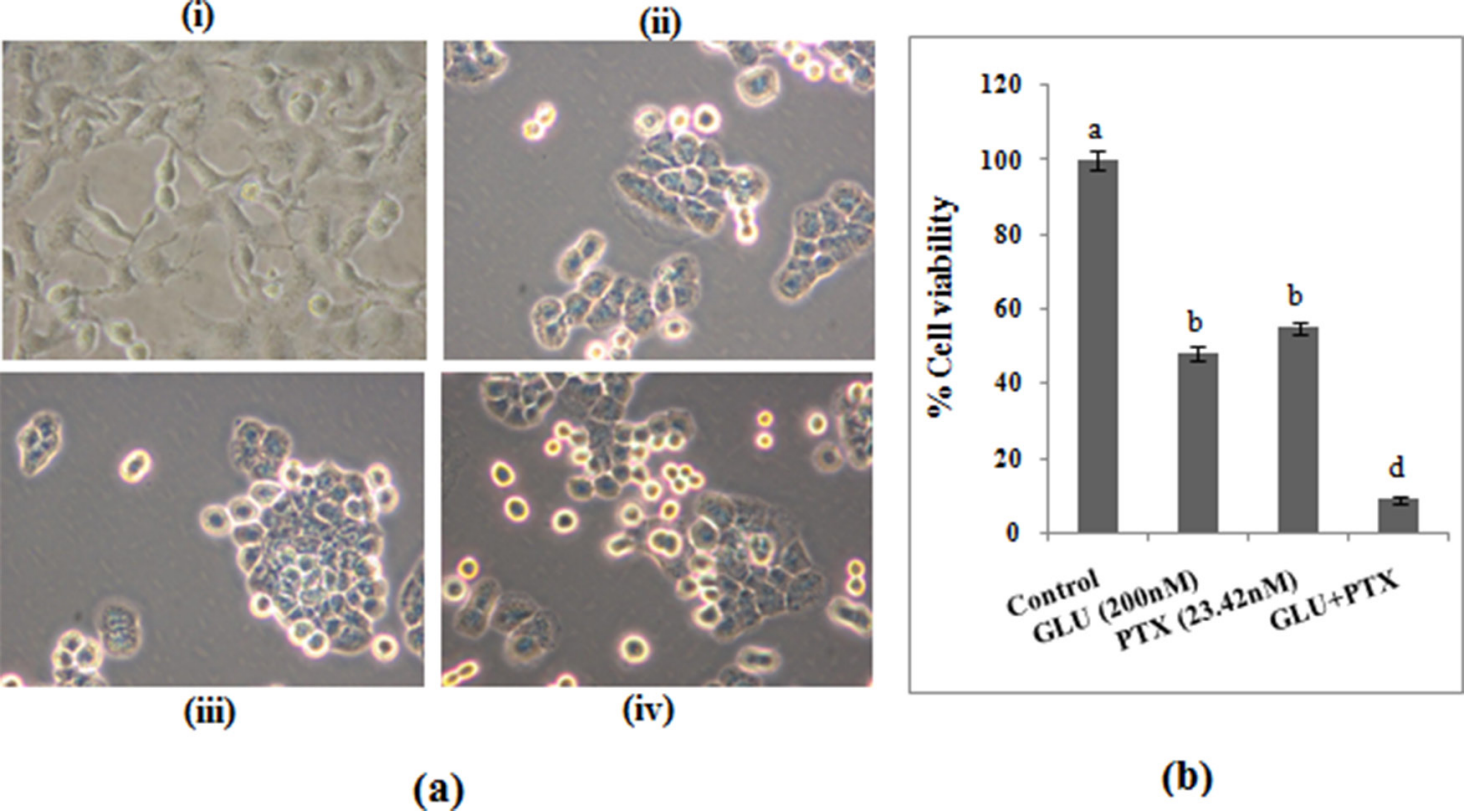
Effect of GLU and PTX on KB cells viability Microscopic images show MTT reduction capability of resistant KB cells. **i.** Control; **ii.** GLU; **iii.** PTX; **iv.** GLU-PTX. Values are given as means ± S.D. of three experiments in each group. Values not sharing a common marking (a, b, c) differ significantly at P ≤ 0.05 (DMRT).

ABCB1 over-expressing resistant KB cells were treated with GLU (200 nM) 1 h before PTX (23.42 nM) exposure and incubated for 24 h. Figure 2a shows photomicrographs of MTT reduction capability of GLU and GLU-PTXin resistant KB cells. Figure 2b shows the effect of GLU, PTX and GLU-PTX on percentage cell viability. It was found that GLU alone treatment showed 56% cell viability in resistant KB cells and PTX alone treatment showed 55% cell viability in sensitive KB cells. The combination of GLU-PTX treatment showed only 8% cell viability of resistant KB cells.

### Effect of GLU-PTX on membrane transport function in resistant KB cells by FACS

**Figure 3a &b:**
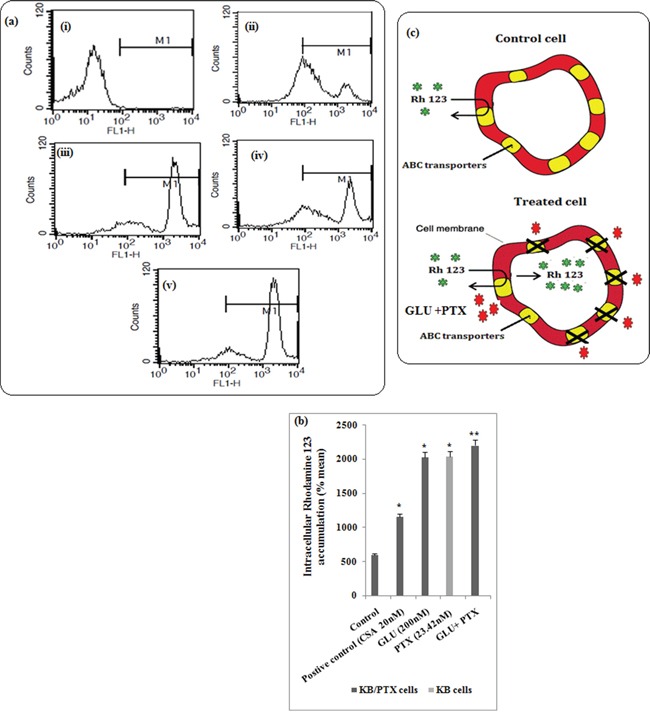
P-gp over expressing resistant KB cells were incubated with 1. Control, 2. Positive Control (Cylosporine A), 3. GLU alone, iv. PTX alone and v. GLU-PTX The level of fluorescence intensity of Rh123 was analyzed by flow cytometry. Data are mean ± S.D from three independent experiments. *P ≤ 0.05 significantly different from control. **c.** Schematic representation of mechanism involved in MDR transport function from cancer cells treated with GLU. In MDR cells, overexpression of MDR transporter proteins increases expulsion of Rh123 from the cell membrane before enzymatic hydrolysis of its esters, thus reducing accumulation of intracellular Rh123. However, GLU inhibit the transport function thereby enhances the accumulation of Rh123 inside the cells.

Flow cytometry measurement of the accumulation of Rh123, a sensitive indicator of P-gp activity, was utilized to examine P-gp (ABCB1) function. In the control cells very minimum Rh123 accumulation was observed due to the efflux of the Rh123 by the transporters. The MDR modulators inhibit the MDR transport function thereby enhance the accumulation of Rh123 inside the cells. An increase in Rh123 accumulation was noted in the cells treated with either cyclosporine A (positive control) or GLU. GLU-PTX combination treatment has shown Rh123 accumulation was increased resistant KB cells compared with PTX alone treated sensitive KB cells, following incubation with Rh123 (120 min) (Figure 3a & 3b).

### Determination of cell cycle by propidium iodide (PI) staining

**Figure 4:**
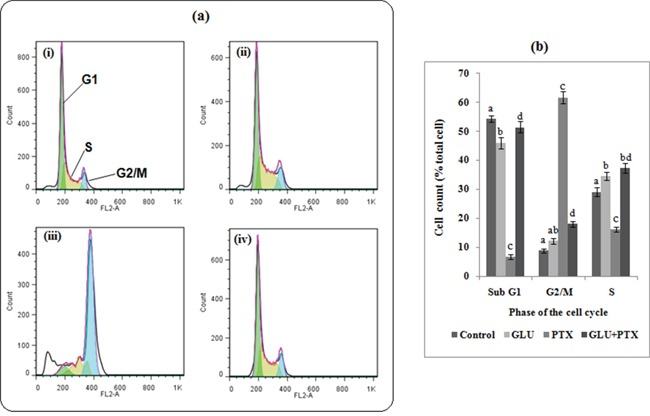
Effect of GLU-PTX on cell cycle and apoptosis **a**. Resistant KB cells were treated with GLU, PTX or GLU-PTX. GLU+ PTX were able to increase the proportion of cells in G2/M as compared to GLU alone ABCB1 expressing resistant KB cells. **b.** The bar graphs show the cell cycle distribution and the percentage of cells in each phase of the cell cycle. Percentage of total cells was obtained by using the CellQuest software.

Cell cycle progression was analyzed to confirm that GLU enhanced the PTX induced cell death efficacy. Resistant KB cells were exposed to GLU, PTX and their combination (24 h) and cell cycle was analyzed by FACS. Greater arrest in G2/M phase observed in sensitive KB cells compared with resistant KB cells, following incubation for 24h. Combination treatment of resistant KB cells with GLU and PTX resulted in an increased trend of cells in S-phase, along with a pronounced arrest in G2/M compared with GLU alone (Figure 4a). No statistical differences in the percentage of G1 phase cells was noted between the combination groups and significant changes was observed in PTX alone treated in sensitive KB cells (Figure 4b).

### GLU-PTX on the expression of ABC transporters proteins in KB cells

**Figure 5:**
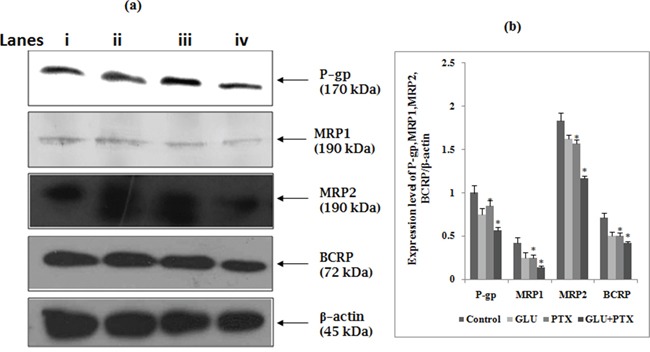
Effect of GLU-PTX on the expression pattern of P-gp-170kDa, MRP1-190kDa, MRP2-190kDa, BCRP-72kDa and β-actin-45kDa expression in KB cells **a.** Lane (i) control, (ii) GLU, (iii) PTX, (iv) GLU-PTX and band intensities were scanned by densitometer (Image ‘J’ software). **b.** The graph represents the P-gp, MRP1, MRP2 and BCRP quantification values normalized to β-actin levels. Densitometric analysis is shown in *p<0.05 vs GLU/PTX group. All the data shown are representative of three independent experiments.

Effect of GLU, PTX and GLU-PTX on the expression pattern of P-gp, MRP1, MRP2 and BCRP in resistant KB cells was analyzed by Western blot (Figure 5a & 5b). The expressions of ABC transporters proteins was found to be decreased in GLU alone or PTX alone treated cells when compared to control cells. GLU-PTX has shown a further decreased in the expression of P-gp, MRP1, MRP2 and BCRP proteins in resistant KB cells, compared to PTX alone treatment in sensitive KB cells.

### GLU-PTX on mRNA expression patterns of ABCB1, BCRP, ABCC1, ABCC2 and ABCC3 in KB cells

**Figure 6:**
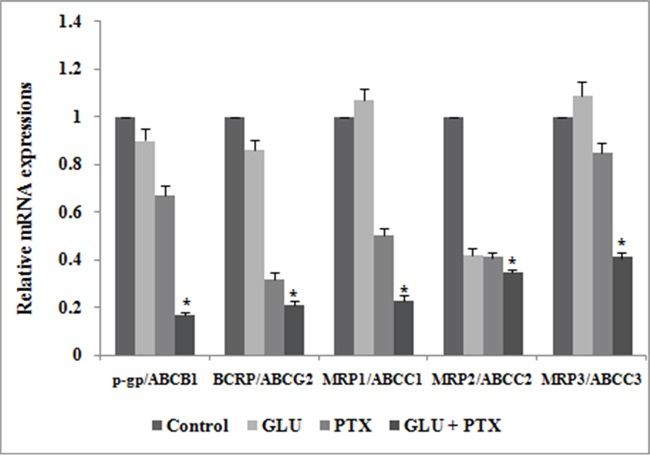
Effect of GLU, PTX and GLU-PTX on relative expression patterns of ABCB1, ABCG2, ABCC1, ABCC2 and ABCC3 in resistant KB cells The delta-delta CT method was used to determine the relative expression patterns of ABCB1, ABCG2, ABCC1, ABCC2 and ABCC3 in resistant KB cells. The values were normalized to the equivalent amounts of 18S rRNA as the reference gene. The data are presented as mean ± S.E. (n=3). *P<0.05 vs the control group.

Figure 6 presents the effect of GLU, PTX and GLU-PTX on the relative mRNA expression levels of ABCB1, BCRP, ABCC1, ABCC2 and ABCC3 genes in resistant KB cells. The mRNA levels of these genes were decreased under GLU alone or PTX alone treatment condition when compared to untreated control group. GLU-PTX combination has resulted in a further decreased of ABCB1, BCRP, ABCC1, ABCC2 and ABCC3 genes in resistant KB cells compared to PTX alone treatment in sensitive KB cells.

### *In silico* molecular interaction of GLU with TMD region of P-gp

We explored the binding affinity (in terms of the docking energy in kcal/mol, docking score and hydrogen bond score) of GLU, a quassinoid to P-gp target. The molecular interaction of GLU (PubChem CID: 441796) with P-gp (PDB ID: 3G61) was analyzed by Schrodinger software (Maestro 9.9) (Figure 7i–7iii). The results were analyzed at the best orientation of the ligand GLU with P-gp and the docking images were documented for representation of ligand-receptor interaction. Initially these molecular interactions were analyzed by sitemap tools. Based on this analysis, we have identified 5 sites of receptor P-gp at which ligand GLU interacts. Among them site 1 and site 2 are considered a major binding affinity with ligand GLU. The sitemap results clearly showed that the P-gp drug ability score for site 1 (1.269) and site 2 (1.057) have a high drug ability to bind with ligand GLU (Table 1).

**Figure 7i:**
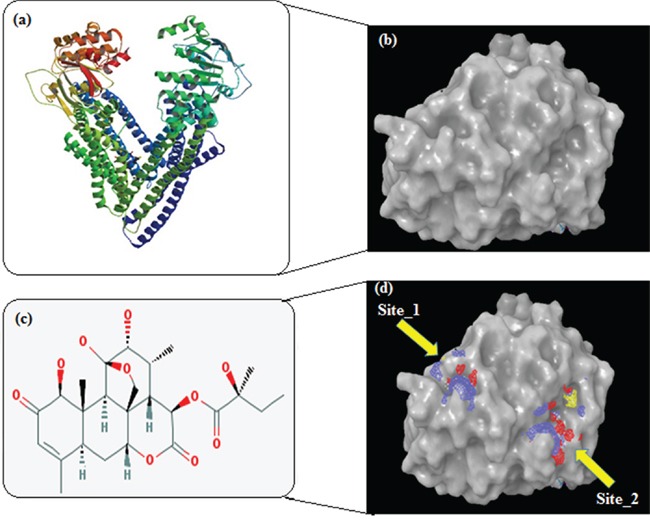
**a.** Homology modelled structure of Human P-gp (PDB ID: 3G61); **b.** Surface moiety structural view of P-gp; **c.** Structure of glaucarubinone (PubChem CID: 441796); **d.** After validation of Ramachandran plot the GLU ligand located different receptor interaction and sitemap identification of the helical transmembrane domains of P-gp by Ligprep application.

**Figure 7ii:**
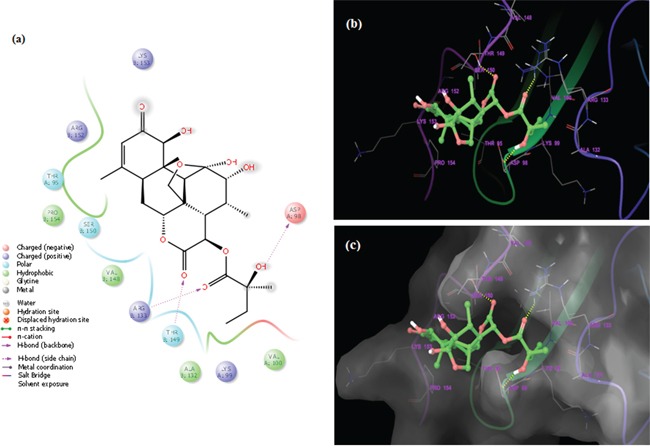
Docked complex of GLU and site-1 homology P-gp by Schrödinger glide application **a.** Ligand interaction diagram; pink lines represent hydrogen bonds, green lines represent π-π interactions. **b.** Structural view; yellow dashed lines indicated hydrogen bonds (Asp98, Arg133 and Thr149). **c.** Structural view; predicted bonded interactions (pink and yellow dashed lines) between GLU and TMD region of P-gp 3D structure.

**Figure 7iii:**
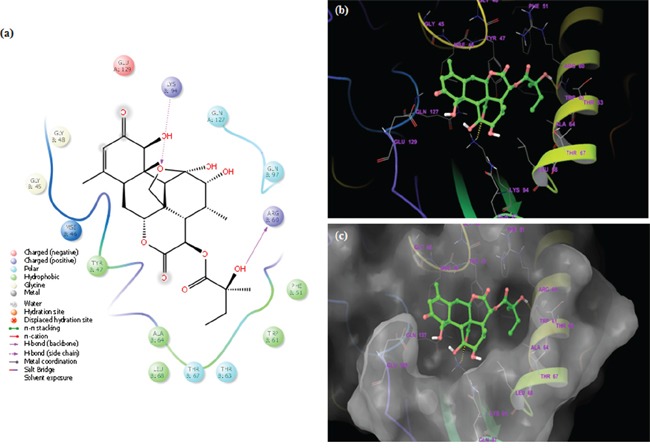
Docked complex of GLU and site-2 homology P-gp by Schrödinger glide application **a.** Ligand interaction diagram; pink lines represent hydrogen bonds, blue and green lines represent π-π interactions. **b.** Structural view; yellow dashed lines indicated hydrogen bonds (Arg60 and Lys94). **c.** Structural view; predicted bonded interactions (pink and yellow dashed lines) between GLU and TMD region of P-gp 3D structure.

**Table 1:** Binding sitemap analysis used as input for receptor grid generation by Schrödinger

Sitemap	Site Score	DScore	Phobic	Philic
Sitemap_site_1	1.166	**1.269**	2.073	0.388
Sitemap_site_2	1.057	**1.057**	1.051	1.062

The binding interactions of GLU were analyzed within site 1 of homology modeled human P-gp by glide docking from Schrödinger. GLU is stabilized through specific interactions such as hydrogen bonding and nonspecific strong interactions such as hydrophobic interactions with ASP98 and THR149 residues in the drug-binding pocket of P-gp (Figure 7ii). At the site 2, it is also observed that ligand GLU forms hydrogen bonding with ARG69 and LYS94 residues, which are located within the helical transmembrane domains of P-gp shown in Figure 7iii. The values of docking score for site 1 (−3.121) and site 2 (−4.324), glide score for site 1 (−3.121) and site 2 (−4324) and hydrogen bond score for site 1 (−1.224) and site 2 (−1.032) indicated that GLU possessed a significant binding affinity with P-gp, thereby indicating that it may inhibit ABC transporters function. Hence, these findings support the choice of GLU as ABC inhibitor to enhance PTX efficacy, in the present study.

### GLU-PTX treatment modulated mRNA expression levels of p53, Bax, Caspase 9, and Bcl-2 by real-time PCR

**Figure 8:**
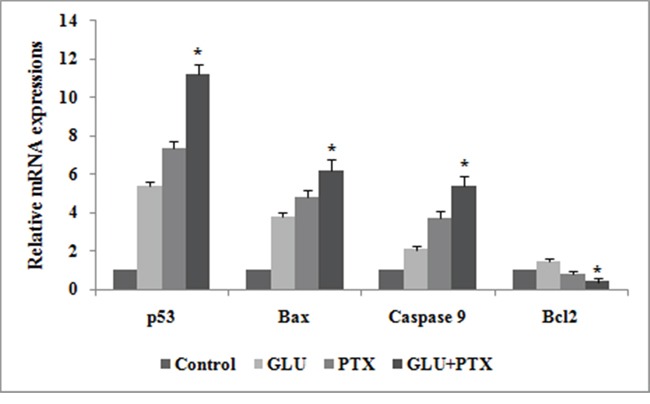
Effect of GLU, PTX and GLU-PTX on relative expression patterns of p53, Bax, Caspase-9 and Bcl-2 in resistant KB cells The delta-delta CT method was used to determine the relative expression patterns of p53, Bax, Caspase-9 and Bcl-2 in resistant KB cells. The values were normalized to the equivalent amounts of 18S rRNA as the reference gene. The data are presented as mean ± S.E. (n=3). *P<0.05 vs the control group.

Figure 8 shows the effects of GLU and GLU-PTX on the relative mRNA expression pattern of p53, Bax, Caspase 9, and Bcl-2 in resistant KB cells. Bax, p53 and Caspase-9 mRNA levels were significantly increased under GLU alone or PTX alone treatment condition when compared to untreated control group. GLU-PTX treated cells showed a further increased mRNA expression of Bax, p53 and Caspase-9 in resistant KB cells compared to GLU treatment group. GLU alone or PTX alone exposure resulted in decreased mRNA expression of Bcl-2 in KB cells. GLU-PTX treated cells showed a further decreased in the mRNA expression of Bcl-2 level in KB cells when compared to PTX alone treatment in sensitive KB cells.

### Effect of GLU-PTX on intracellular ROS generation in resistant KB cells

**Figure 9:**
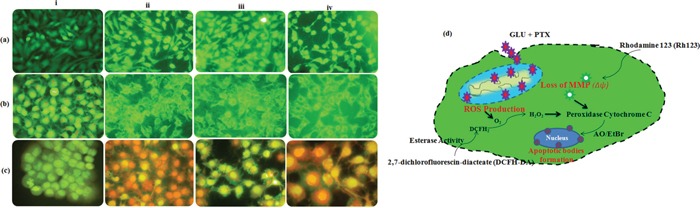
Fluorescence photomicrographs show the effect of GLU-PTX on **a.** intracellular ROS generation by DCFH-DA staining; **b.** mitochondrial membrane potential by Rhodamine 123 and **c.** apoptotic morphology changes by EtBr and AO staining in resistant KB cells. i. Control; ii. GLU; iii. PTX; iv. GLU-PTX. **d.** Schematic representation of mechanism involved in ROS overproduction, mitochondrial dysfunction (ΔΨm) and apoptotic bodies' formation could initiate the pre-apoptotic responses from KB cells treated with GLU-PTX.

**Figure 10:**
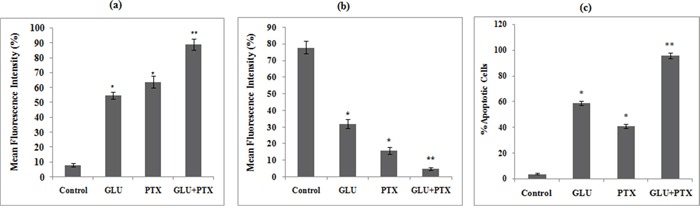
**a.** Release of mean florescence intensity ROS (%) from GLU-PTX treated KB cells for 24 h. **b.** The alteration in mitochondria membrane potential of GLU-PTX treated KB cells. **c.** Percentage of apoptosis in KB cells. Number of apoptotic and viable cells in the microscopic field were counted and % apoptotic cells calculated. Values are given as means ± S.D. of six experiments in each group. (*) p ≤ 0.05 and (**) p ≤ 0.01, compared with untreated cells (control groups).

Intracellular ROS level was measured by using a non-fluorescent probe, 2,7-diacetyl dichlorofluorescein (DCFH-DA). ROS generation were observed in GLU, PTX and GLU-PTX treated in ABCB1 over expressing resistant KB cells. While weak ROS generation was observed in the untreated resistant KB cells. GLU alone and PTX alone treated cells showed significant ROS production. But enhanced ROS generation was found in GLU-PTX treated resistant KB cells (Figure 9a). The percentage of mean fluorescence intensity values confirms the DCF fluorescence in different treatment groups (Figure 10a).

### GLU-PTX modulates mitochondrial membrane potential in KB cells

Fluorescence microscopic images showed accumulation of Rh123 dye in the control group. No Rh123 accumulation was found in GLU-PTX treated cells as the membrane potential decreased (Figure 9b). The percentage of mean fluorescence intensity values confirms the mitochondrial membrane potential was found to be reduced in the GLU-PTX treated cells when compared with GLU alone and PTX alone treated cells (Figure 10b).

### Observation of chromatin condensation

Apoptotic features with condensed or fragmented chromatin, indicative of apoptosis, were observed in GLU, PTXand GLU-PTX treated in resistant KB cells. Control cells showed evenly distributed acridine orange stain (green fluorescence) with no morphological changes whereas GLU, PTX and GLU-PTX treated cells showed apoptotic morphological features indicated by the presence of Ethidium bromide fluorescence inside the cells due to membrane damage (Figure 9c). GLU-PTX treatment showed 98% of apoptotic cells in ABCB1 over expressing resistant KB cells whereas GLU treatment showed only 58% apoptotic cells and PTX treatment showed 43% apoptotic cells (Figure 10c).

### Detection of DNA fragmentation in resistant KB cells

**Figure 11:**
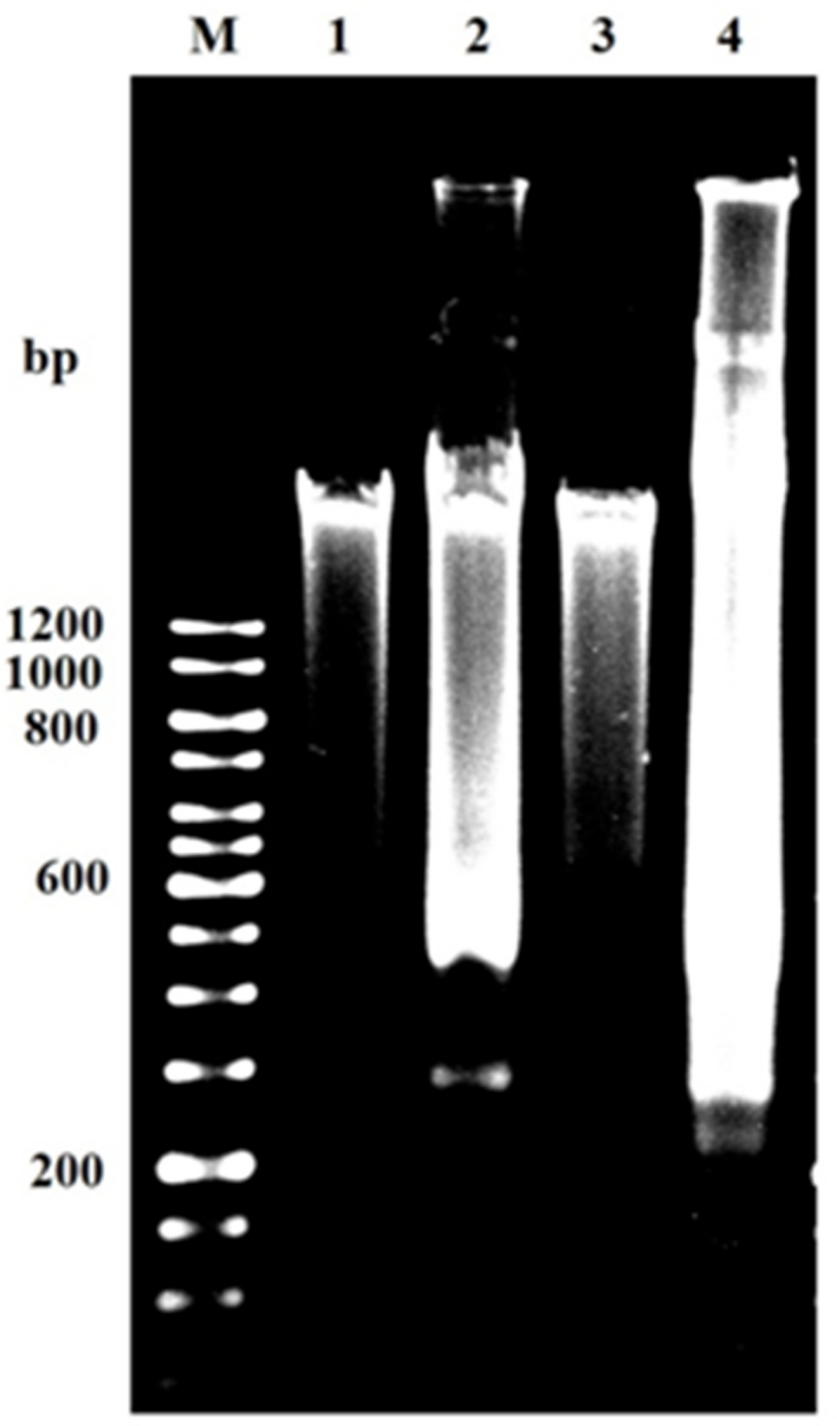
Apoptotic DNA fragmentation by gel electrophoresis showing DNA ladder of GLU alone and GLU-PTX combination treated resistant KB cells for 24 h M: 12 kb DNA markers; 1: control (untreated cells). 2: GLU alone 3: PTX and 4: GLU-PTX. The results are representative of three independent experiments carried out in the same conditions. DNA ladder formation indicates apoptosis as seen in lanes 2–4.

Agarose gel electrophoresis of DNA from GLU and GLU-PTX treated ABCB1 over expressing resistant KB cells showed a ladder-like pattern of DNA fragments consisting of approximately 180–200 base pairs. Photographs of the gels are shown in Figure 11. DNA fragmentation was observed in GLU-PTX in resistant KB cells. A significant increase in the 180–200 bp oligonucleosomal DNA fragments was found in the GLU-PTX treated group compared to PTX alone treatment in sensitive KB cells. In comparison, the DNA from untreated cells did not exhibit any significant fragmentation or smearing.

### Protective effect of glu and its combinations in human normal blood lymphocytes (HBL)

#### Effect of GLU on normal lymphocytes

**Figure 12:**
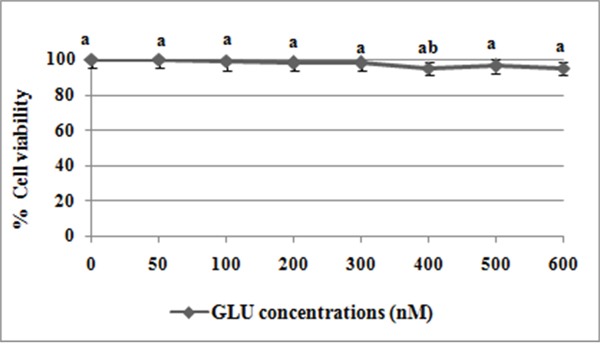
Effect of different concentrations of GLU on lymphocytes viability Values are given as means ± S.D. of three experiments in each group. Values not sharing a common marking (a, b, c) differ significantly at *P* ≤ 0.05 (DMRT).

Figure 12 shows the effect of different concentrations of GLU on % cell viability of blood lymphocytes. GLU treatment at concentrations 0-600 nM has not affected the % viability of lymphocytes at the end of 24 h incubation.

### GLU-PTX on cell viability in human peripheral blood lymphocytes

**Figure 13:**
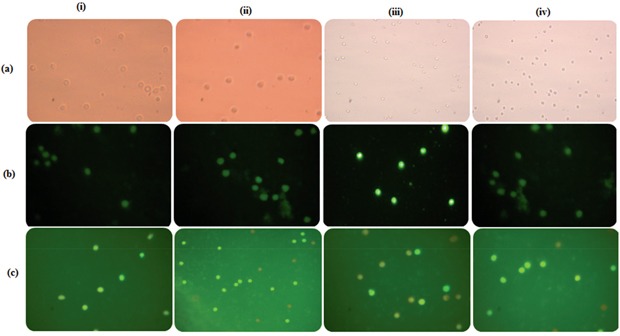
**a.** Microscopic images show MTT reduction capability of human blood lymphocytes (HBL). Purple colored crystal formation is directly proportional to cell viability; **b.** GLU-PTX on the intracellular ROS generation in HBL cells by DCFH-DA staining; **c.** apoptotic morphology changes by EtBr and AO staining in HBL cells. There was no apoptotic features were observed during treatment with GLU-PTX in human blood lymphocytes. i. Control; ii. GLU; iii. PTX; iv. GLU-PTX.

**Figure 14:**
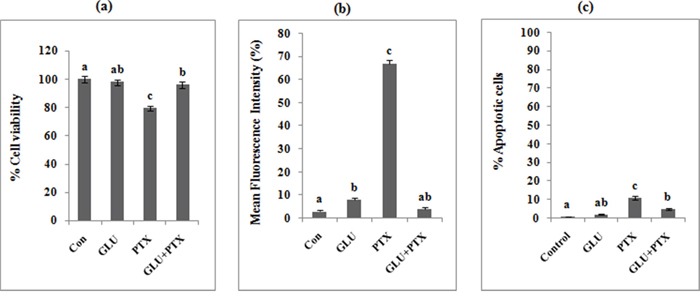
**a.** Percentage cell viability in GLU, PTX and GLU-PTX treated HBL cells. **b.** Release of mean florescence intensity ROS (%) from GLU-PTX treated HBL cells for 24 h. **c.** Percentage of apoptosis in HBL cells. Number of apoptotic and viable cells in the microscopic field were counted and % apoptotic cells calculated. Values are given as means ± S.D. of three experiments in each group. Values not sharing a common marking (a, b, c) differ significantly at *P* ≤ 0.05 (DMRT).

GLU alone treatment at concentrations 200 nM has not affected the % viability of lymphocytes (Figure 13a) at the end of 24 h incubation. PTX treatment at conc. 23.42 nM showed a significant toxicity in normal lymphocytes. A combination treatment did not show any significant change in lymphocyte viability (Figure 14a).

### Effect of GLU-PTX on intracellular ROS generation changes in HBL by DCFH-DA staining method

PTX treatment at conc. 23.42 nM showed a significant increase in the DCF fluorescence thus indicating ROS generation in normal lymphocytes (Figure 13b). There was no significant increase of DCF fluorescence during treatment with GLU-PTX in human blood lymphocytes. The percentage of mean fluorescence intensity values confirms there was no significant DCF fluorescence in different treatment groups (Figure 14b).

### Effect of GLU-PTX on apoptotic morphological changes in human peripheral blood lymphocytes

Figure 13c shows the photomicrographs of apoptotic morphological changes in different treatment groups by EtBr and AO staining. The control cells show evenly distributed green fluorescence (AO stain). PTX alone treatment showed a significant apoptotic morphological changes in human blood lymphocytes but no significant apoptotic features were detected in treatment with GLU alone and GLU-PTX in human blood lymphocytes. Number of apoptotic and viable cells in the microscopic fields were counted and % apoptotic cells calculated. There was no significant change in % apoptotic cells in the control, GLU and GLU-PTX treated cells, compared to PTX alone treatment (Figure 14c).

## DISCUSSION

Quassinoids are well-known as the bitter principles of *Simaroubacaeous* plants. They are highly oxygenated triterpenes and possess a broad range of both *in vitro* and *in vivo* biological activities [15, 5]. GLU is a quassinoid known to occur in S*imaroubaceae* plants. It was first isolated from the seeds of the tree *Simarouba glauca*, and originally developed as an antimalarial drug [6], and has more recently been recognised as an anticancer agent. Its anticancer activity has been demonstrated against breast, prostate and, ovarian cancer [7, 8] and importantly its effects on drug-resistant KB cells and also on P-gp expression have not yet to be elucidated. On the other hand, PTX and other anthracylines are commonly used in the treatment of a wide range of cancers, especially against oral, non-small cell lung cancer, metastatic breast cancer and refractory ovarian cancer [16]. PTX works by interfering with normal microtubule breakdown during cell division, leading to cellular apoptotic death [17, 4]. The major problem with clinical use of PTX is the frequent development of MDR, which hampers its efficacy for cancer treatment [12]. Therefore, assessing the drug resistant properties of cancer cells and the reversal of the resistance properties are important steps in the development of a successful treatment. Further, blockade or inhibition of the ABC transporters may prove to be novel strategy to overcome chemoresistance. In the present study, we demonstrated for the first time that GLU was used as an adjuvant to enhance the chemosensitivity of PTX, and to reverse the MDR in KB oral cancer cells. We propose that ROS production, including altering the MMP, after GLU and/or PTX treatments significantly influences the initiation of the cellular mechanisms involved in inhibiting MDR transporters proteins and in inducing apoptosis. Therefore, the cytotoxicity of PTX in resistant KB cells was improved when it was combined with GLU.

Our results show that GLU (200 nM) treatment 1 h before to PTX (23.42 nM) exposure greatly inhibited the KB cells proliferation. The present study also illustrated the superior anticancer effect of GLU-PTX combination than PTX alone or GLU alone treatment. Similar to our present findings, resveratrol, a polyphenol from red wine, interacts with P-gp and also inhibits cell growth and enhances the cytotoxicity of vincristine, paclitaxel and adriamycin in KBv200 and NCI-H60 lung cancer cells [4]. In addition, previous studies reported that nilotinib significantly enhanced the cytotoxicity of the ABCB1 substrates colchicine, vinblastine and paclitaxel in KB-C2 and KB-V1 cells [11]. These results are consistent with the finding that auraptene in grapefruit juice and nobiletin in valencia orange juice increased the cytotoxicity of traditional chemotherapeutic agents in the human cervical carcinoma cell line KB-V1 [18]. Based on the pervious findings, our results clearly indicated that these concentrations could be sufficient to inhibit the functions of P-gp and other ABC transporters like MRPs and BCRP.

As P-gp functions as an efflux pump of many anticancer drugs, the selected KB cells proving increased P-gp expression pumped out PTX from the cells into the extracellular medium, thereby parading a resistance to PTX-induced apoptotic cell death. Flow cytometry measurement of the accumulation of Rh123, a sensitive indicator of P-gp activity, was utilized to examine P-gp function [4]. Our result showed that the cellular accumulation of Rh123 was increased with GLU and PTX combination treatment. The increase in the accumulation of Rh123 in drug-resistant cells after the addition of GLU indicates that this compound causes a modulation of resistance by inhibiting the pump function of P-gp and other transporters such as MRPs and BCRP. Consistent with our results, previous studies have indicated that the combination of phenolics, terpenoids and alkaloids increase the accumulation of the Rh123 and calcein-AM in CEM/ADR5000 cells. These results are also comparable to those of verapamil (positive control), which also inhibits P-gp activity [19]. In addition, pervious study has shown that resveratrol increased the intracellular concentrations of doxorubicin in acute myeloid leukemia (AML) cells by inhibiting membrane transport function [20]. Curcumin is also reported to increase the Rh123 accumulation and inhibit the function of P-gp ATPase activity in KB-V1 cells [21]. The pervious findings also support the present investigation, which indicated a high accumulation of doxorubicin within HL60/MDR cells treated with *Schisandra chinensis* lignans [22] and natural lignans from *Arctium lappa* seeds, namely arctigenin, matairesinol and, arctiin can increase the retention of the P-gp substrate Rh123 in CEM/ADR cells, indicating that these lignans can inhibit the activity of P-gp and, other ABC transporters [23].

It has been reported that PTX exerts cytotoxicity by destabilizing microtubules and inhibiting depolymerization back to tubulin, resulting in mitotic inhibition and ultimately causes arrest cells in the G2/M phase [17]. Previously, we have shown that the response to PTX is augmented by resveratrol in ABCB1-overexpressing NCI-H60 non-small cell lung carcinoma [4]. Drug-induced G2/M arrest is associated with double-strand DNA damage, extensive chromosome condensation and finally induces cell death through an apoptotic pathway [24]. Our data showed that GLU could increase cytotoxicity of PTX in KB cells at relatively lower doses, which may be due to enhanced intracellular PTX accumulation. Moreover, cell cycle analysis confirmed that combination treatment with GLU and PTX resulted in a increased trend of cells in S-phase, along with a pronounced arrest in G2/M phase compared with GLU alone. Since the cell cycle arrest in the G2/M phase is usually a cellular response to PTX treatment, it can be assumed that GLU potentiate the effect of PTX in resistant KB cells. A previous report has suggested that *S. chinensis* lignans in combination with doxorubicin significantly increased the proportion of G2/M phase in HL60/MDR cells [22]. Docetaxel is a microtubule inhibitor affecting the S and G2/M phases of the cell cycle [25]. In addition, docetaxel combined with fractionated extracts of *Curcuma wenyujin* and *Chrysanthemum indicum* could enhance the apoptosis and also owned the ability to induce G2/M phase arrest in both MCF-7/ADR and A549/Taxol cells [26]. One of the most important mechanisms of active drug efflux is mediated by the classical MDR is attributed to the elevated expression of P-gp, MRPs, and BCRP [2]. We observed that the combined treatment of GLU-PTX significantly decreased the expression level of P-gp, MRPs, and BCRP proteins in resistant KB cells. Similar to our present findings, resveratrol increased the susceptibility of the doxorubicin-resistant cells to apoptotic cell death and decreased the expression levels of MRP1 in acute myeloid leukemia-2/DX300 cells at both mRNA and protein levels [20]. Moreover, in this study, GLU-PTX treatment further significantly decreased the mRNA expression levels of ABCB1, BCRP, ABCC1 ABCC2 and ABCC3 in KB cells. Overexpression of P-gp is the most frequent event in causing multidrug resistance. Recent report showed that resveratrol inhibits genistein-induced MRP2 protein synthesis and its mRNA expression in HepG2 cells [27]. In this study, GLU sensitized KB cells to PTX action by inhibiting ABC transporters specified gene expressions. Therefore, decreasing the expressions of ABC transporter may be involved in the chemosensitizing effect of GLU in oral cancer cells, especially for the treatment of MDR human carcinoma. Our previous observations on lung cancer cells NCI-H460 overexpressing ABC transporter proteins (LRP, ABCB1, BCRP, ABCC1 ABCC2 and ABCC3) confirmed the ability of resveratrol lignans to overcome the resistance to PTX [4]. Further, the results corroborate with previous report as the combinations of digitonin with secondary metabolites (phenolics, terpenoids and alkaloids) resulted in a significantly decreased the expressions of P-gp. These results provide evidence that the selected secondary metabolite interfere directly and/or indirectly with P-gp function [19]. Furthermore, it has been reported that quercetin can inhibit the expression of P-gp activity and decreased this transporter in EPG85-257RDB cells at both mRNA and protein levels [28].

Although GLU is not a substrate of P-gp and/or MRPs, it is able to inhibit the transport of other P-gp substrates and inhibit drug resistance. Resistance of cancer cells has also been attributed to the cell-surface receptors. To increase deeper visible into cellular and molecular modes of action of GLU, we performed for the first time *in silico* molecular docking studies of GLU to P-gp receptors by Schrodinger (Mastero 9.9). The results were analyzed at the best orientation of the ligand GLU with P-gp and the docking images were documented for representation of ligand-receptor interaction. The values of docking score, glide score and hydrogen bond score indicated that GLU possessed a significant binding affinity with P-gp, thereby indicating that it may inhibit ABC transporters function. Interestingly, the results of these *in silico* docking studies indeed indicated that site-1 and 2 to be the preferable binding site for GLU within the drug-binding pocket of human P-gp. These results may be taken as a clue that GLU kills P-gp expressing, drug-resistant selected KB cells by binding to P-gp at their active sites leading to interruption in signaling cascades, thereby, inhibition of tumor growth and induction of cell death. This point of view is supported by ours experimental evidence that GLU as P-gp inhibitor to enhance PTX efficacy, in the present study.

The increase of ROS production that can induce oxidative stress and inhibit P-gp functions and other ABCs expressions in resistant cells [29, 30]. However, the relationship between the effect of GLU on ROS production and inhibition of P-gp and MRPs has not been reported. Our study provides important evidence that MDR transporter-mediated PTX resistance was inhibited. Thus, GLU enhances the intracellular accumulation of PTX and Rh123 (substrates of P-gp and MRP efflux pump) through the decrease in mRNA and protein expression of P-gp, MRPs and BCRP. We used this functional assay to verify the involvement of MDR transporters in PTX-resistance and the potential role of GLU as a MDR modulator. Elevated ROS levels can induce DNA damage, thereby activating p53 dependent apoptotic cascade [3]. Our study shows that the intracellularly accumulated GLU possibly interacts with peroxidase-H_2_O_2_ system and significantly generates ROS production in resistant KB cells. Thus, GLU acts as pro-oxidant, disrupting intracellular redox balance and leading to cancer cell apoptosis [8]. On the other hand, PTX is a substrate of P-gp and MRPs that potently stimulates the ATPase activity of P-gp [4]. Compounds that are effluxed by MDR transporters may deplete ATP, and the offer of ATP from ADP by oxidative phosphorylation produces ROS. We recommend that the generation of ROS by PTX could exhibit a further effect on cellular oxidation-reduction imbalance and cause oxidative stress on KB cells, triggering additional MDR transporters to pump PTX out and resulting in resistance development. However, the decrease in mRNA expression of MDR transporters by GLU through ROS production reversed the resistance and increased the cytotoxicity and cell death elicited by PTX. Although the data supported the involvement of ROS within the mechanism by which GLU interrupted transporter expression, the elaborated signal transduction pathways need further investigation.

**Scheme 1:**
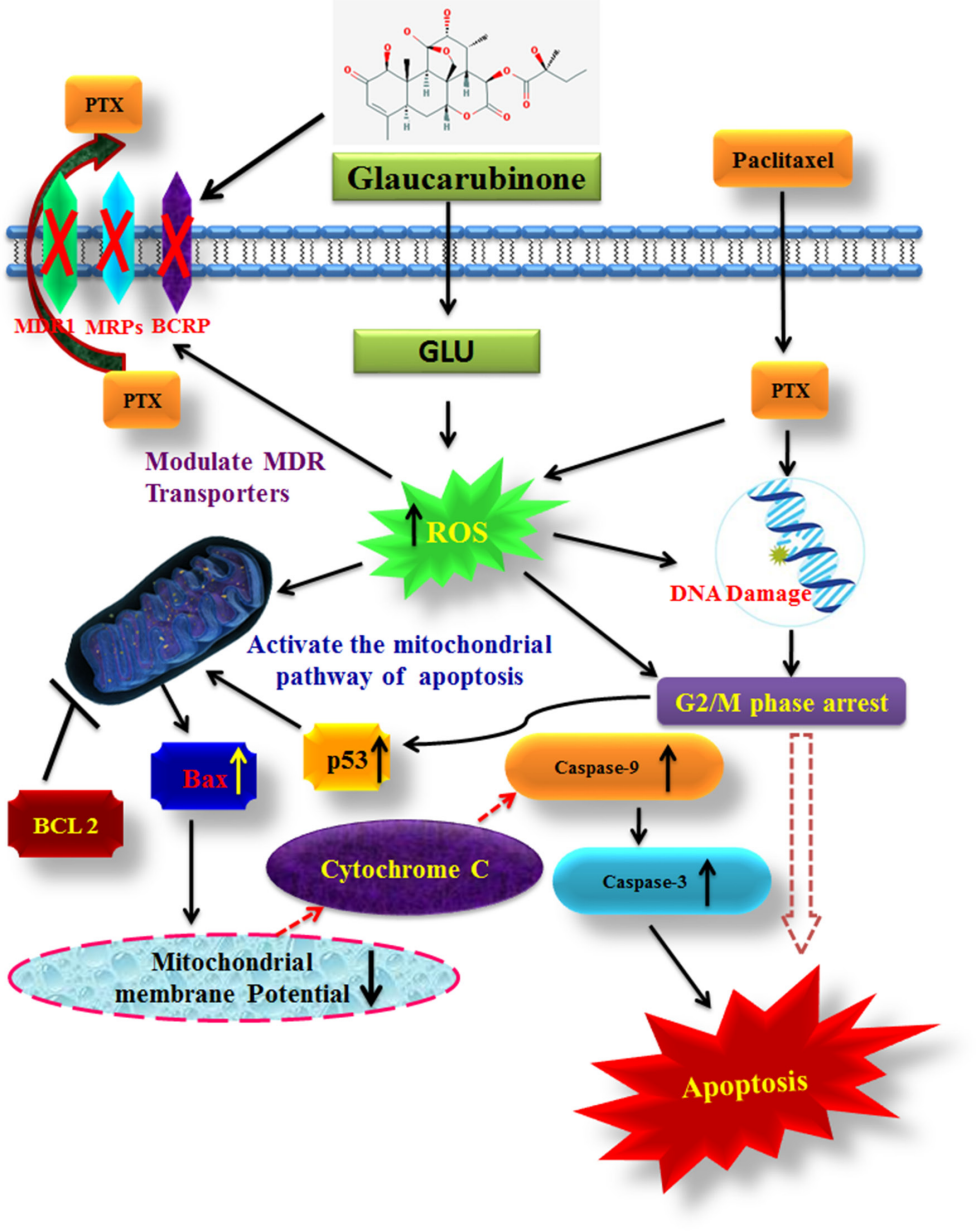
The proposed schematic illustration for the possible mechanisms of reversing pump and non-pump MDR by GLU and/or PTX in KB cells

In present study, we tend to provided three evidences that the inhibitory effect of GLU on cell growth is due to apoptosis. Based on our results, we found that GLU can activate cell death via induction of p53, Bax, and Caspase-9 is understood to activate Bid, a Bcl-2 family protein, which leading to the activation of caspase-9, enhancing release of cytochrome c by mitochondria. In addition, previous studies reported that caspase-3 is activated via many routes, including through the activity of caspase-9, caspase-8 and Bid, which seem to work along together to amplify apoptotic signals [31]. Thus, activation of caspase-9 via GLU is probably going to amplify the apoptotic signal via both the caspase-8 and mitochondrial pathways, which might account for the proficient induction of apoptosis we observed GLU treated in resistant KB cells. Results were additionally confirmed by the characteristic morphological features of apoptosis such as chromatin condensation, cytoplasm shrinkage, nuclear fragmentation, nucleolus pyknosis, and apoptotic bodies in KB cells stained with AO/EtBr after treatment with GLU or GLU-PTX. The characteristic of apoptosis was also further confirmed by determination of the DNA ladder formation which is a result of combined GLU and PTX treatment elicited significant apoptosis causing effect as proved by markedly clear DNA ladder formation. Loss of mitochondrial potential is a beginning stage of apoptosis. Mitochondrion is one of the chief organelles in regulating apoptosis [32]. Apoptosis induced by GLU occurred in a mitochondrial-relevant manner. In the present study, it has also been observed that an increase of disruption of mitochondrial membrane potential is considered to be an indicator of mitochondria damage and normally is defined as an early stage of apoptosis, preceding outflow of tiny molecules from the mitochondria (including cytochrome c, apoptosis-inducing factor, etc.) and followed by caspase-9/caspase-3 cascade activation [33]. Our results showed that GLU-PTX treated cells showed a further increased MMP alteration in KB cells than GLU treatment alone. Therefore, our data clearly imply that the elevated ROS production can induce DNA damage, thereby activating p53 dependent apoptotic cascade by GLU contributed to the decrease in the MDR transporter expression. The results corroborate with previous reports as the 4-hydroxy-2,6-dimethoxychalcone from *Polygonum limbatum* eisn, strongly induced apoptosis as via the MMP disruption as well as increase in ROS level in adriamycin-resistant human acute T-lymphoblastic CEM/ADR5000 leukemia cells [29]. To the best of our knowledge, the present study innovated in indicating that the membrane damage allowed GLU influx and induced intracellular PTX overload, which further changed the levels of ROS production and variation of MMP; modulated the expression and function of ABC transporters. All these complex oxidative stresses ultimately induced apoptosis through the over expression of pro-apoptotic proteins via the intrinsic mitochondrial pathway in KB cells. We examined, at least in part, our results with GLU and PTX indicate that the proposed pathways for reversing pump and non-pump MDRs in KB cells are closely associated (Scheme 1). While cancer cells endure complex stress, these relationships are obligatory to verify the proliferative (ABC transporter-mediated MDR resistance) or death (intrinsic mitochondrial mediated apoptotic pathways) of cancer cells.

Despite the eminent role of chemotherapy, its achievement is limited by the development of drug resistance and severe side effects [34]. Additionally, the present study also focussed on investigation of the protective effect of GLU and combination drugs in human normal blood lymphocytes. In order to find out whether GLU has any cytotoxic effect on normal lymphocytes, lymphocytes were alienated into 6 groups and treated with different concentrations of GLU (0-600nM). The effect of GLU on different experiments ((a) percentage cell viability, (b) determination of intracellular ROS, and (c) apoptotic morphological changes) were analysed in normal human blood lymphocytes. Further, we observed GLU treatment at concentrations (0-600 nM) has not impacted the % viability of lymphocytes at the end of 24 h incubation. Particularly, GLU alone treatment at concentrations 200 nM has not impacted the % viability of lymphocytes. PTX treatment at conc. 23.42 nM showed a significant toxicity in normal lymphocytes. The combination treatment (GLU-PTX) did not show any significant change in lymphocyte viability. Furthermore, the combination treatment showed that there was no significant ROS production and apoptotic morphological features in human blood lymphocytes. These results clearly indicated that GLU is able to induce selective toxicity in cancer cells.

In conclusion, as GLU is a naturally occurring phytochemical that is present in a wide variety of Simaroubacaeous plants, the results suggest that GLU may prove useful as a subsidiary dietary supplement for patients with oral carcinoma who are resistant to chemotherapeutic drugs, especially for PTX. The present study concluded that GLU enhances the cytotoxicity of PTX resistant KB cells, which is consistent with the increased intracellular accumulation of the drug. Further, we show that GLU induces G2/M arrest of PTX-resistant human oral cancer cells. Furthermore, GLU-PTX treatment further significantly decreased the mRNA expression levels of ABCB1, BCRP, ABCC1, ABCC2 and ABCC3 in KB cells. Similarly, the protein level of ABCB1, BCRP and MRPs was decreased during GLU-PTX treatment. Additionally, *in silico* molecular docking studies revealed that GLU possess greater binding affinity with trans-membrane domain region of P-gp/ABCB1. The effect is mainly exerted on the cells by induction of apoptosis, which is consequent with the differently altered activities of pro-apoptotic family members, particularly, the production of ROS, DNA fragmentation, apoptotic morphological features, loss of mitochondrial membrane potential, and the activation of p53, Bax, and caspase-9. Taken together, on one hand the reversal effect of GLU on multidrug resistance of KB cells can be made by downregulating such as P-gp, MRPs and BCRP. On the other hand, the GLU can induce apoptosis via p53, Bax, and caspase-9 activation, which was confirmed by the fact that incubation of KB cells with GLU caused an increase of PTX-induced apoptosis. Finally, we strongly suggest that GLU may be a promising compound, especially for the treatment of MDR human carcinoma and the toxicological and preclinical studies of GLU using animal models are envisaged.

## MATERIALS AND METHODS

### Chemicals

Glaucarubinone was kindly provided by Dr. John A. Beutler, National Cancer Institute (NCI), Chemotherapeutics Repository, Molecular Targets Laboratory, Frederick, USA. Cyclosporine-A (CSA), Paclitaxel, 3-(4,5-dimethylthiazol-2-yl)-2,5-diphenyl tetrazolium bromide (MTT), 2,7-dichlorofluorescindiacteate (DCFH-DA), Rhodamine 123 (Rh123), Heat inactivated fetal calf serum (FCS), RPMI-1640 medium, Glutamine-Penicillin-Streptomycin solution, Histopaque-1077, Trypsin-EDTA were purchased from Sigma Chemicals Co., St. Louis, USA. qRT-PCR and RT-PCR primers were purchased from Integrated DNA Technologies, Inc. USA. Takyon^TM^ No Rox SYBR MasterMix dTTP blue was purchased from Eurogentec, Belgium. Monoclonal rabbit antibodies for MDR1/ABCB1, ABCG2/BCRP, MRP1/ABCC1, β-actin and goat anti-rabbit IgG-HRP polyclonal antibody were purchased from Cell Signalling Technology, USA. Analytical grade acetone, ethanol, dimethyl sulfoxide (DMSO) were bought from SRL, India.

### Maintenance of KB oral cancer cells

KB, human oral squamous carcinoma cell line was procured from National Center for Cell Science (NCCS), Pune, India. The cells were grown as monolayers in RPMI-1640 medium supplemented with 10% FCS, 1 mM Sodium pyruvate, 10 mM HEPES, 1.5 g/L Sodium bicarbonate, 2 mM L-glutamine and antibiotics (10,000 U/mL Pencillin and 10 mg/mL Streptomycin). Stocks were maintained in 75 cm^2^ tissue culture flasks in a humidified atmosphere of 5% CO_2_ and 95% air at 37°C. Cultures were maintained in the medium until the confluent growth. Each experimental culture was performed with culture density of 1 × 10^6^ cells.

### Development of paclitaxel resistant KB cells

Multidrug resistant oral KB cells highly express a classical membrane transporter, P-glycoprotein (ABCB1). In the present work, ABCB1 overexpressing resistant KB cells were established by culturing cells (sensitive) with increasing concentration of PTX in the medium to 1, 1.5, 2, 2.5, 3, 3.5, and 4 μM. Cells were grown for 1 week at each concentration of PTX. To confirm this, the inhibition of cell growth on increasing PTX concentrations was examined in KB (sensitive) and KB/PTX (resistant) cells by MTT assay.

### Treatment of KB cells

Stock solutions of glaucarubinone (GLU) and paclitaxel (PTX) (1 mg/mL) were prepared in 0.5% dimethyl sulphoxide (DMSO) and stored at 4°C. Further dilution was made in culture media to obtain the desired concentrations. The final concentrations of DMSO in the culture medium were not more than 0.01% (v/v). 0.01% DMSO was used as a sham control.

### Dose fixation and cell growth inhibition evaluation by MTT assay

Sensitive KB cells (5 × 10^3^ cells/well) were treated with different concentration of PTX (0-70 nM) and resistant KB cells (5 × 10^3^ cells/well) with GLU (0-600 nM) and cytotoxicity was observed after 24 h at 37°C, at the end of the incubation period, MTT (5 mg/mL in PBS) solution was added to each well and allowed to develop color for additional 4 h. Plates were placed on a plate shaker for 30 min and read immediately at 570 nm using an enzyme-linked immunosorbent assay reader. Experiments were performed in triplicate. IC_50_ values were calculated and the optimum dose was used for the chemosensitizing experiments. Sham control was run with resistant KB cells that received 0.01% DMSO. For chemosensitization experiments, resistant KB cells were treated with GLU (200 nM) 1 h before PTX (23.42 nM) treatment.

### Experimental protocol for chemosensitizing experiments

Resistant KB cells were divided into four groups as follows. Each group contained the cell density of 1 × 10^6^.

**Table:** 

Group 1	:	Resistant KB cells (untreated cells)
Group 2	:	Resistant KB cells + GLU (200 nM)
Group 3	:	Sensitive KB cells + PTX (23.42 nM)
Group 4	:	Resistant KB cells + GLU (200 nM) + PTX (23.42 nM)

### Measurement of ABC transporters functional assay in KB cells

The accumulation of PTX was determined by Rh123, a fluorescent substrate of P-gp in cancer cells as has been previously described. Briefly, resistant KB cells (2×10^5^ per sample) were treated with GLU, PTX and GLU-PTX (or 20 nM Cyclosporine A as positive control) for 60 min and then incubated with 20 μM Rh123 for 1 h (in the dark, 37°C in a 5% CO_2_). Following Rh123 accumulation, cells were washed twice with ice-cold PBS. Fluorescence intensity of Rh123 in individual cells was measured immediately by a FACScalibur excitation at 488 nm and emission at 530 ± 15 nm (BD Biosciences, San Jose, CA).

### Determination of cell cycle by propidium iodide (PI) staining

Resistant KB cells (2×10^5^) seeded on the 6-well plates were treated with GLU, PTX and their combination at 37°C for 24 h. For analyzing DNA content, 2 x10^5^cells were fixed in 90% ethanol in PBS at 4°C. After 12 h, the fixed cells were pelleted, gently resuspended in ice cold PBS and stained with 0.5 mg/mL PI plus 50 μg/mL RNAase. The samples were then incubated at 37°C for 30 min, stored in the dark at 4°C. The red fluorescence of the individual cells was measured at an excitation wavelength of 540 nm and an emission wavelength at 610 nm in a FACScaliber flow cytometer (BD Biosciences, San Jose, CA). A minimum of 10,000 events were analyzed per sample using CellQuest software.

### Western blot analysis

KB cells were cultured in 60 mm dishes at 60% confluency prior to drug treatment. Cells were treated with GLU-PTX at desired concentration. Whole cell proteins were extracted with RIPA lysis buffer (150 mM NaCl, 0.5% Triton X-100, 50 mM Tris–HCl, pH 7.4, 25 mM NaF, 20 mM EGTA, 1 mM DTT, 1 mM Na3VO4, 0.1% SDS, and protease inhibitors cocktail) for 30 min on ice followed by centrifugation at 12,000 rpm for 15 min. The protein concentration of the supernatant was measured by using the BCA reagent. Proteins (30-40 μg) were electrophoresed on 12% gradient Tris–HCl gel (Bio-Rad, semi-dry, USA) and electrotransferred onto polyvinylidene difluoride (PVDF) membrane in Tris–glycine buffer (pH 8.4) containing 20% methanol. The membrane was then blocked in 5% fat-free dry milk in phosphate-buffered saline with 0.1% Tween-20 (PBS-T) for 1 h. Membranes were probed with monoclonal rabbit anti-ABCB1, ABCC1, ABCC2 and BCRP antibodies and horseradish peroxidase-conjugated secondary antibody by standard western blot procedures. Then, the membranes were washed with TBST thrice for 10 min interval, after extensive washes in TBST, the bands were visualized by treating the membranes with 3, 3′-diaminobenzidine tetrahydrochloride/H_2_O_2_ for localization and enhanced Luminate Forte Western HRP substrate detection reagent. Densitometry was done using ‘Image J’ analysis software.

### mRNA studies of the ABC transporters and pro/anti-apoptotic genes

The total RNA was extracted from the KB cells using RNeasy Mini kit (Analytic Jena, Germany) as per the protocol recommended by the manufacturer. The mRNA expression of ABC transporters genes (ABCB1, BCRP, ABCC1, ABCC2, ABCC3) and pro/anti apoptotic genes (p53, Bax, Caspase-9, Bcl-2) in KB cells was determined using real-time PCR and the primer sequences are given in Table 2. Samples were run in triplicate to ensure amplification integrity. Manufacturer-supplied (Integrated DNA Technology (IDT), USA) primer pairs were used to measure the mRNA expression. Quantitative RT-PCR (qRT-PCR) was performed using Takon No Rox SYBR MasterMix (Eurogentec, Belgium) on a Thermal cycler CFX96 QRT-PCR system (Bio-Rad, CFX96, USA). The thermal cycles for PCR amplification were carried out with a carry over prevention at 50°C for 3 min and Takyon mix activation at 95°C for 3 min followed by 40 cycles of denaturation (95°C for 10 s), annealing (60°C for 20 s) and extension (72°C for 30 s) as recommended by the manufacturer (Eurogentec, Belgium). The expression levels of genes were normalized to the expression level of the 18S mRNA in each sample. The threshold for positivity of real-time PCR was determined based on negative controls. The calculations for determining the relative level of gene expression were made using the cycle threshold (Ct) method. The mean Ct values from triplicate measurements were used to calculate the expression of the target gene using the 2^−ΔΔCt^ formula.

**Table 2:** List of primer sequence

Gene name	Primer sequence
*ABCB1*	F: 5′ TGGAGGTAAGTGACCCAGGGCTG 3′ R: 5′ AGGCAATCCGATGCAGAGCCCA 3′
*ABCG2*	F: 5′ TGTAAAACGACGGCCAGT 3′ R: 5′ CAGGAAACAGCTATGACC 3′
*ABCC1*	F: 5′ GTGGCTATCAAGGGCTCCGTGG 3′ R: 5′ TCCGCGTCTTCTCGCCAATCT 3′
*ABCC2*	F: 5′ GTCTTCGTTCCAGACGCAGTCC 3′ R: 5′ CACGTGGAGAAGCTGCCAGGG 3′
*ABCC3*	F: 5′ GGCATGGCCAGGGCTCATTGG 3′ R: 5′ GGTCCACGTACACGTACACCCA 3′
*p53*	F: 5′ GAGAATCTCCGCAAGAAAGG 3′ R: 5′ CTCATTCAGCTCTCGGAACA 3′
*Bax*	F: 5′ GGGCCCACCAGCTCTGA 3′ R: 5′ CCTGCTCGATCCTGGATGA 3′
*Caspase-9*	F: 5′ TGCTGAGCAGCGAGCTGTT 3′ R: 5′ AGCCTGCCCGCTGGAT 3′
*Bcl-2*	F: 5′ CTTGACAGAGGATCATGCTGTAC 3′ R: 5′ GGATGCTTTATTTCATGAGGC 3′
*18S rRNA*	F: 5′ AGGAATTCCCAGTAAGTGCG 3′ R: 5′ GCCTCACTAAACCATCCAA 3′

### Molecular docking studies

The 3D structure of the P-gp was downloaded from Protein Data Bank (PDB) and modified. The modification included removal of water molecules from the cavity, stabilizing charges, filling in the missing residues, generation of side chains etc, according to the parameters available. After modification the transporter was biologically active and stable. Using chemdraw software the structure of the drugs and analogs were sketched and generated the MOL structure. GLU was docked at each of the generated grids (sites 1 to 4 and the ATP binding site of P-gp by using the “Extra Precision” (XP) mode of Glide program v5.0 (Maestro 9.9, Schrödinger, Inc., New York) with the default functions. The top scoring ligand's conformation was used for graphical analysis. All computations were carried out on a Toshiba Satellite (C80-I5010) core i3 processor with Linux OS.

### Determination of intracellular ROS levels

Intracellular ROS level was measured using a non-fluorescent probe, 2,7-diacetyl dichlorofluorescein (DCFH-DA), that penetrates into the intracellular matrix of cells where it is oxidized by ROS to fluorescent dichlorofluorescein (DCF). GLU-PTX and free GLU treated resistant KB cells were seeded in 6 well plates (2×10^5^ cells/well) and incubated with 10 μM DCFH-DA for 30 min at 37°C. The cells were observed under fluorescence microscope using blue filter (450-490 nm) (Olympus, CKX41, Japan).

### Changes in mitochondrial transmembrane potential (Δψ)

The changes in mitochondrial membrane potential during GLU and GLU-PTX treatment condition were analyzed using Rh123 staining. GLU and GLU-PTX treated cells were mixed with 1 μL of Rh123 (5 mmol/L) and kept incubation for 15 min. Then, the cells were washed with PBS and observed under fluorescence microscope using blue filter (450-490 nm) (Olympus, CKX41, Japan).

### Observation of chromatin condensation using a fluorescence microscope

Acridine orange (AO) and Ethidium bromide (EtBr) dual staining method was adopted to differentiate condensed apoptotic nuclei from normal cells. The control, GLU and GLU-PTX treated resistant KB cells were seeded in 6 well plate (2 × 10^5^ cells/well) and incubated in a CO_2_ incubator for 24 h. The cells were stained with 1:1 ratio of AO/EBr, and observed under a fluorescence microscope with a magnification of 40X. The number of cells showing features of apoptosis was counted as a function of the total number of cells present in the field.

### Detection of DNA fragmentation by agarose gel electrophoresis

Resistant KB cells were cultured in 60 mm dishes to 70% confluency prior to drug treatment. Cells (density at 5×10^6^) were treated with control, GLU and PTX and GLU-PTX at desired concentration for 24 h. After incubation cells were harvested by centrifugation at 2000×g for 5 min and washed once with ice cold PBS (Kubota 2420, Tokyo). The genomic DNA was extracted from the resistant KB cells using QIAamp DNA Mini Kit (Qiagen, USA) as per the protocol recommended by the manufacturer. Samples were electrophoresed in 0.9% (w/v) agarose gel. The gel was stained with EtBr (1 μg/mL) and photographed using gel documentation system (Gene Flash, Syngene, Bioimaging). The presence of apoptosis was indicated by the appearance of a ladder of oligonucleosomal DNA fragments on the agarose gel.

### Experimental design for lymphocyte study

The isolation of human blood lymphocytes was done using standard procedure. Briefly, blood samples were aseptically collected in heparinized sterile tubes. Lymphocytes were isolated using Ficoll–histopaque (Sigma, USA). Blood was diluted 1:1 with PBS and layered onto Histopaque at the ratio of blood and PBS:Histopaque maintained at 4:3. The blood was centrifuged at 1340 rpm for 35 min at room temperature. The lymphocyte layer was removed and washed twice in PBS at 1200 rpm for 10 min each, and then washed with (RPMI-1640) media. The number of lymphocytes was counted using a haemocytometer and the viability of the cells was assayed by the trypan blue exclusion test. Approximately 2×10^5^ cells were present in 1.0 mL lymphocyte suspension. In order to find out whether GLU has any cytotoxic effect on normal lymphocytes, lymphocytes were divided into 6 groups and treated with different concentrations of GLU (0-600nM). The effect of GLU on different parameters ((a) percentage cell viability, (b) determination of intracellular ROS, and (c) Apoptotic morphological changes) were analysed in normal human blood lymphocytes.

### Statistical analysis

Data are expressed as means ± standard error of the mean (SE). Statistical differences were determined by one-way analysis of variance (ANOVA). ‘P’ values less than 0.05 were considered significant.
